# The effectiveness of vaccination to prevent the papillomavirus infection: a systematic review and meta-analysis

**DOI:** 10.1017/S0950268818003679

**Published:** 2019-03-20

**Authors:** Herney Andrés García-Perdomo, Julio Cesar Osorio, Adrian Fernandez, James Alejandro Zapata-Copete, Andrés Castillo

**Affiliations:** 1 Universidad del Valle, Cali, Colombia; 2 UROGIV Research Group, Universidad del Valle, Cali, Colombia; 3 School of Basic Sciences at Universidad del Valle, Cali, Colombia; 4 School of Public Health at Universidad del Valle, Cali, Colombia; 5 Department of Epidemiology at Universidad Libre, Cali, Colombia; 6 Department of Biology, Faculty of Natural and Exact Sciences at Universidad del Valle, Cali, Colombia

**Keywords:** Human papillomavirus, meta-analysis, systematic review, vaccine

## Abstract

Our purpose was to determine the effectiveness and harms of vaccination in patients with any sexual history to prevent the prevalence of papillomavirus infection. A search strategy was conducted in the MEDLINE, CENTRAL, EMBASE and LILACS databases. Searches were also conducted in other databases and unpublished literature. The risk of bias was evaluated with the Cochrane Collaboration's tool. Analysis of fixed effects was conducted. The primary outcome was the infection by any and each human papillomavirus (HPV) genotype, serious adverse effects and short-term adverse effects. The measure of the effect was the risk difference (RD) with a 95% confidence interval (CI). The planned interventions were bivalent vaccine/tetravalent/nonavalent *vs.* placebo/no intervention/other vaccines. We included 29 studies described in 35 publications. Bivalent HPV vaccine offers protection against HPV16 (RD −0.05, 95% CI −0.098 to −0.0032), HPV18 (RD −0.03, 95% CI −0.062 to −0.0004) and HPV16/18 genotypes (RD of −0.1, 95% CI −0.16 to −0.04). On the other side, tetravalent HPV vaccine offered protection against HPV6 (RD of −0.0500, 95% CI −0.0963 to −0.0230), HPV11 (RD −0.0198, 95% CI −0.0310 to −0.0085). Also, against HPV16 (RD of −0.0608, 95% CI −0.1126 to −0.0091) and HPV18 (RD of −0.0200, 95% CI −0.0408 to −0.0123). There was a reduction in the prevalence of HPV16, 18 and 16/18 genotypes when applying the bivalent vaccine, with no increase in adverse effects. Regarding the tetravalent vaccine, we found a reduction in the prevalence of HPV6, 11, 16 and 18 genotypes, with no increase in adverse effects.

## Introduction

The development of the three FDA-approved multivalent prophylactic human papillomavirus (HPV) vaccines has been because of the discovery of HPV infection as the cause of all cervical cancer [1]. The three prophylactic HPV vaccines have shown high efficacy for prevention of HPV infection [2].

Cervarix^®^ and Gardasil^®^ were the first vaccines for the prevention of the cervical cancer. Cervarix^®^ targets types 16 and 18 HPV, which are responsible for 70% of all cervical cancer [3], while Gardasil^®^, on the other hand, adds also activity against types 6 and 11 HPV, which cause 90% of anogenital warts [4]. Additionally to those included in the qHPV, the 9vHPV vaccine contains type 31, 33, 45, 52 and 58 antigens [5].

These vaccines are well-tolerated, safe and only with minor adverse effects. They also protect against pre-cancerous lesions caused by subtypes 16 and 18 in a naive population, adding a systemic immune response at 5 years post-vaccination [6–8].

The primary care service in United States recommended HPV vaccine for females aged 9–26, through the Vaccines for Children program since 2006 [9]. Additionally, the immunisation for adolescents aged 11–12 years and for adolescent women (aged 13–26 years) before sexually active was recommended by The US Center for Disease Control and it is accepted in many developed countries [7,10].

According to the health care system's organisation, the coverage rate and the programmes differ. Some of the vaccination programmes are offered through schools (e.g. Australia, UK) whereas others are provided in private clinics, or public primary care (e.g. the United States) [11,12].

Different studies (phases II and III) have been conducted, and a few reviews have tried to pooled the effects [13], however, none of them emphasise the changes in the prevalence/incidence of HPV serotypes in the populations. Besides, previous reports have not assessed the impact of vaccination in different ages and time since sexual beginning, nor multisite and cross-protection against HPV types. Also, there is conflict about the protection against re-infection among individuals known to be infected and who subsequently clear their infections [14].

The objective was to determine the effectiveness and harms of vaccination in patients with any sexual history to prevent the prevalence of papillomavirus infection.

## Methods

We performed this review according to the recommendations of the Cochrane Collaboration [15] and following the PRISMA Statement [16]. The PROSPERO registration number is CRD42017074007.

### Eligibility criteria

We included only clinical trials; both gender patients from any population at any age and any sexual history. The interventions were: vaccine against HPV: HPV16 and HPV18 (bivalent vaccine), or HPV6, HPV11, HPV16 and HPV18 (tetravalent vaccine) and HPV6, HPV11, HPV16, HPV18, HPV31, HPV33, HPV45, HPV52 and HPV58 (nanovalent) genotypes. The vaccination had to be intramuscular injection over a period of 6 months according to the standard scheme. We did not include pregnant patients

The comparisons were: bivalent vaccine/tetravalent/nanovalent *vs.* placebo/no intervention/other vaccines.

*Outcomes*:

- Infection by any HPV genotype: assessment must be by any validated technique identified by the blood sample and oral cell sample. If patients are older than 18 years old, the sample must come from the specific organ.
- Infection by each HPV genotype: assessment must be by any validated technique identified by the blood sample and oral cell sample. If patients are older than 18 years old, the sample must come from the specific organ.
- Serious adverse effects.
- Short- and long-term adverse effects.

For infection and long-term adverse effect outcomes, studies had at least 1-year follow-up and for short-term outcome studies were allowed to be less than a 1-year follow-up.

### Information sources

A literature search was conducted as recommended by Cochrane. We used medical subject headings (MeSh), Emtree language, Decs and text words related. We searched MEDLINE (OVID), EMBASE, LILACS and the Cochrane Central Register of Controlled Trials (CENTRAL). The search was performed from 2000 to to date.

To ensure literature saturation, we scanned references from relevant articles identified through the search; conferences (HPV2015 and HPV2017) related to HPV and vaccines and thesis databases (e.g. Theseo). Grey literature was searched in open grey and Google scholar. We looked for clinical trials in the process in clinicaltrials.gov, the registry created by the World Health Organization and the New Zealand registry for Clinical trials, among others. We contacted authors by e-mail in case of missing information. There were no setting or language restrictions.

### Data collection

Two researchers reviewed each reference by title and abstract and full-texts, applied pre-specified inclusion and exclusion criteria. Disagreements were resolved by consensus and where the dispute could not be solved, a third reviewer dissolved conflict.

Two trained reviewers using a standardised form independently extracted the following information from each article: study design, geographic location, authors names, title, objectives, inclusion and exclusion criteria. Also, the number of patients, type of laboratory technique, losses to follow-up, timing, definitions of outcomes, outcomes and association measures and funding source.

### Risk of bias

We assessed the risk of bias for each study with the Cochrane Collaboration tool, which covers: sequence generation, allocation concealment, blinding, incomplete outcome data, selective reporting and other biases. Two independent researchers will judge about the possible risk of bias from extracted information, rated as ‘high risk,’ ‘low risk’ or ‘unclear risk.’ We described a graphic representation of potential bias using RevMan 5.3 [17].

### Data analysis/synthesis of results

We performed the statistical analysis in R [18]. For categorical outcomes, we reported information in risk differences (RD) with 95% confidence intervals (CIs) according to the type of variables, and we pooled the data with a random effect meta-analysis according to the heterogeneity expected. The results were reported in forest plots. Heterogeneity was evaluated using the *I*^2^ test [15]. For the interpretation, it was determined that the values of less than 50% would be for low heterogeneity and more than 50% would be for the high level of heterogeneity.

### Publication bias

We did not perform publication bias due to the number of included studies in each meta-analysis.

### Sensitivity analysis

We performed sensitivity analysis extracting weighted studies, analysing the length of follow-up and running the estimated effect to find differences.

### Subgroup analysis

We tried to perform subgroup analysis by co-infection, sample site, gender, age and continent however due to the scarcity of data, we were not able to do it.

## Results

**Fig. 1.**
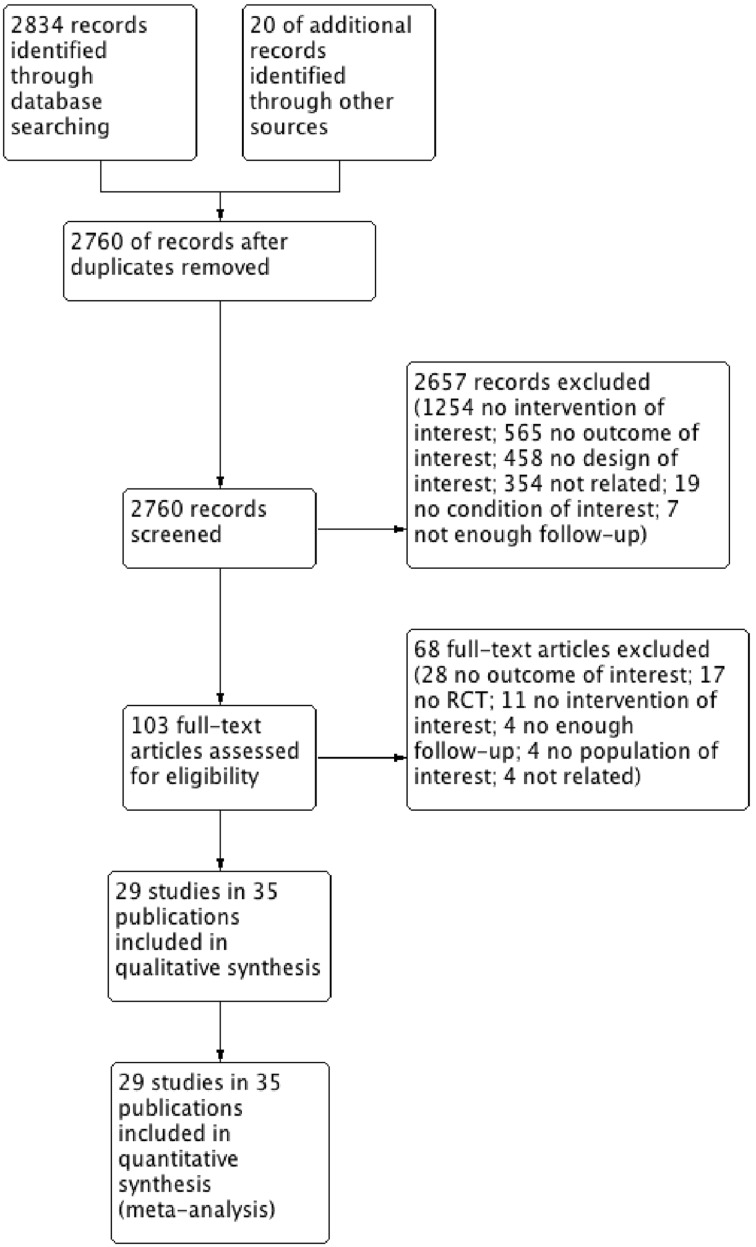
Flowchart for selected studies.

We found 2834 records through the electronic search strategy and 20 through other searches (Fig. 1). After excluding duplicates, we ultimately included 29 unique studies described in 35 publications in our qualitative and quantitative analysis (Kim 2010 [19], Harper 2006 [20], Kang 2008 [21], Reisinger 2007 [22], Levin 2010 [23], Giuliano 2011 [24], Muñoz 2009 [25] (including Castellsague 2011 [26]), Villa 2006 [27] (including Villa 2005 [28]), Rivera Medina 2010 [29], Vesikari 2015 [30], Konno 2010 [31], Lehtinen 2016 [32], Moreira Jr 2011 [33], Salif Sow 2013 [34], Wheeler 2008 [35], Herrero 2013 [36] (including Beachler 2016 [37], Hildesheim 2014 [38] and Herrero 2011 [39]), Lazcano-Ponce 2009 [40], Kim 2011 [41], Mugo 2015 [42], Wheeler 2016 [43], Zhu 2014 [44], Olsson 2007 [45], Apter 2015 [46] (including Szarewski 2012 [47] and Palmroth 2012 [48]), Toft 2014 [49], Einstein 2014 [50], Schmeink 2011 [51], Konno 2014 [52], Naud 2014 [53], Lim 2014 [54]).

### Included studies

#### Settings

Most of the studies were conducted in several countries. Only Wheeler 2008 (United States), Kang 2007 (Korea), Lazcano-Ponce 2009 (Mexico), Kim 2010 (Korea), Levin 2010 (United States), Konno 2010 (Japan), Kim 2011 (Korea), Schmeink 2011 (Netherlands), Herrero 2013 (Costa Rica), Konno 2014 (Japan), Zhu 2014 (China), Lim 2014 (Malaysia), Toft 2014 (Denmark) and Lehtinen 2016 (United States) conducted their studies in a single country.

#### Comparisons

Harper 2006, Kim 2010, Rivera Medina 2010, Konno 2010, Kim 2011, Schmeink 2011, Salif Sow 2013, Herrero 2013, Einstein 2014, Konno 2014, Zhu 2014, Lim 2014, Toft 2014, Naud 2014, Apter 2015, Lehtinen 2016 and Wheeler 2016 used the bivalent vaccine as the primary intervention. The rest of the studies used the tetravalent vaccine, and the only one that used the 9vHPV vaccine was Vesikari 2015.

#### Gender

All studies included data from females. However, there were four studies (Reisinger 2007, Moreira Jr 2011, Giuliano 2011 and Lehtinen 2016) described information for males.

The rest of the information regarding the included studies is described in Table 1.

**Table 1.** Characteristics of included studies

Author, year	Country	Age	Gender	Intervention	Control	Follow-up	*N*
Harper, 2006	Canada, United States and Brazil	15–25	Female	HPV16/18 vaccine	Placebo	53 months	606
Villa 2006 (Villa, 2005)^a^	Brazil, Finland, Sweden, Norway and United States	16–23	Female	HPV6/11/16/18 vaccine	Placebo	5 years	468
Wheeler, 2008	United States	16–23	Female	HPV6/11/16/18 vaccine	HBV Vaccine	2,5 months	3754
Olsson, 2007	Brazil, Finland, Sweden, Norway and United States	16–23	Female	HPV6/11/16/18 vaccine	Placebo	5 years	552
Reisinger, 2007	Countries of Europe, Asia and the Americas	9–15	Male and female	HPV6/11/16/18 vaccine	Placebo	18 months	1749
Kang, 2008	Korea	9–23	Female	HPV6/11/16/18 vaccine	Placebo	7 months	176
Lazcano-Ponce, 2009	Mexico	18–23	Female	HPV6/11/16/18 vaccine	Placebo	4 years	679
Muñoz, 2009 (Castellsagué, 2011)^a^	Colombia, France, Germany, Philippines, Spain, Thailand and United States	24–45	Female	HPV6/11/16/18 vaccine	Placebo	4 years	2817
Kim, 2010	Korea	10–14	Female	HPV16/18 vaccine	HAV vaccine	7 months	957
Rivera Medina, 2010	Australia, Colombia, the Czech Republic, France, Germany, Honduras, Korea, Norway, Panama, Spain, Sweden and Taiwan	10–14	Female	HPV16/18 vaccine	HAV vaccine	12 months	2067
Levin, 2010	United States	7–12	Female	HPV6/11/16/18 vaccine	Placebo	14 days	126
Konno, 2010	Japan	20–25	Female	HPV16/18 vaccine	HAV vaccine	2 years	877
Kim, 2011	Korea	15–25	Female	HPV16/18 vaccine	Placebo	7 days	225
Moreira Jr, 2011	18 countries	16–26	Males	HPV6/11/16/18 vaccine	Placebo	3 years	4049
Giuliano, 2011	18 countries	16–26	Males	HPV6/11/16/18 vaccine	Placebo	3 years	4059
Schmeink, 2011	Netherlands	9–15	Female	HPV16/18 vaccine	HBV vaccine	12 months	494
Salif Sow, 2013	Senegal and Tanzania	10–25	Female	HPV16/18 vaccine	Placebo	12 months	1941
Herrero, 2013 (Beachler, 2016; Hildesheim, 2014; Herrero, 2011)^a^	Costa Rica	18–25	Female	HPV16/18 vaccine	HAV vaccine	4 years	5834
Einstein, 2014	NA	18–45	Female	HPV16/18 vaccine	HPV6/11/16/18 vaccine	5 years	1106
Konno, 2014	Japan	20–25	Female	HPV16/18 vaccine	HAV vaccine	4 years	867
Zhu, 2014	China	18–25	Female	HPV16/18 vaccine	Placebo	4 years	5972
Lim, 2014	Malaysia	18–35	Female	HPV16/18 vaccine	Placebo	15 months	271
Toft, 2014	Denmark		Female	HPV16/18 vaccine	HPV6/11/16/18 vaccine	12 months	92
Naud, 2014	Brazil, Canada and United States	15–25	Female	HPV16/18 vaccine	Placebo	9,4 years	431
Vesikari, 2015	Belgium, Denmark, Finland, Italy, Spain and Sweden	9–15	Female	9vHPV vaccine	HPV6/11/16/18 vaccine	7 months	599
Mugo, 2015	Ghana, Kenya and Senegal	9–26	Female	HPV6/11/16/18 vaccine	Placebo	15 days	NA
Apter, 2015 (Szarewski, 2012; Palmroth, 2012)^a^	Countries of Europe, Asia-Pacific and the Americas	15–25	Female	HPV16/18 vaccine	HAV vaccine	12 months	18 644
Lehtinen, 2016	United States	12–16	Male and female	HPV16/18 vaccine	HBV vaccine	12 months	32 176
Wheeler, 2016	Countries of Europe, Asia-Pacific and the Americas	>25 years	Female	HPV16/18 vaccine	Placebo	84 months	4747

a Studies with other publications.

Studies with follow-up less than 1 year, were included for adverse effects.

### Excluded studies

Sixty-eight full-text articles were excluded (28 studies did not have the outcome of interest; 17 were not RCT; 11 studies had no intervention of interest; four studies did not have enough follow-up; four had no population of interest, and four were not related).

### Risk of bias assessment

We found that 50% and 75% of the included studies were graded as unclear on describing the random sequence generation (Einstein 2014; Giuliano 2011; Harper 2006; Herrero 2013; Konno 2010; Konno 2014; Lehtinen 2016; Levin 2010; Moreira Jr 2011; Mugo 2015; Muñoz 2009; Naud 2014; Olsson 2007; Villa 2006) and the allocation concealment (Einstein 2014; Giuliano 2011; Harper 2006; Kang 2008; Kim 2010; Kim 2011; Konno 2010; Konno 2014; Lazcano-Ponce 2009; Lehtinen 2016; Levin 2010; Lim 2014; Mugo 2015; Naud 2014; Olsson 2007; Reisinger 2007; Rivera Medina 2010; Schmeink 2011; Villa 2006; Wheeler 2016; Zhu 2014) respectively.

Most of the studies described the blinding of participants and personnel appropriately. Kim 2010; Konno 2010; Konno 2014; Lehtinen 2016; Levin 2010; Naud 2014; Vesikari 2015; Villa 2006 and Wheeler 2008 were graded as unclear. Only one study (Schmeink 2011) was graded as high risk of bias since it was unblinded. Regarding the outcome assessment, only Villa 2006 was graded as unclear.

Apter 2015; Giuliano 2011; Harper 2006; Kim 2010; Kim 2011; Lazcano-Ponce 2009; Lim 2014; Naud 2014, Villa 2006; Wheeler 2008 and Salif Sow 2013 were graded as high risk of bias due to the high proportion of lost to follow-up.

**Fig. 2.**
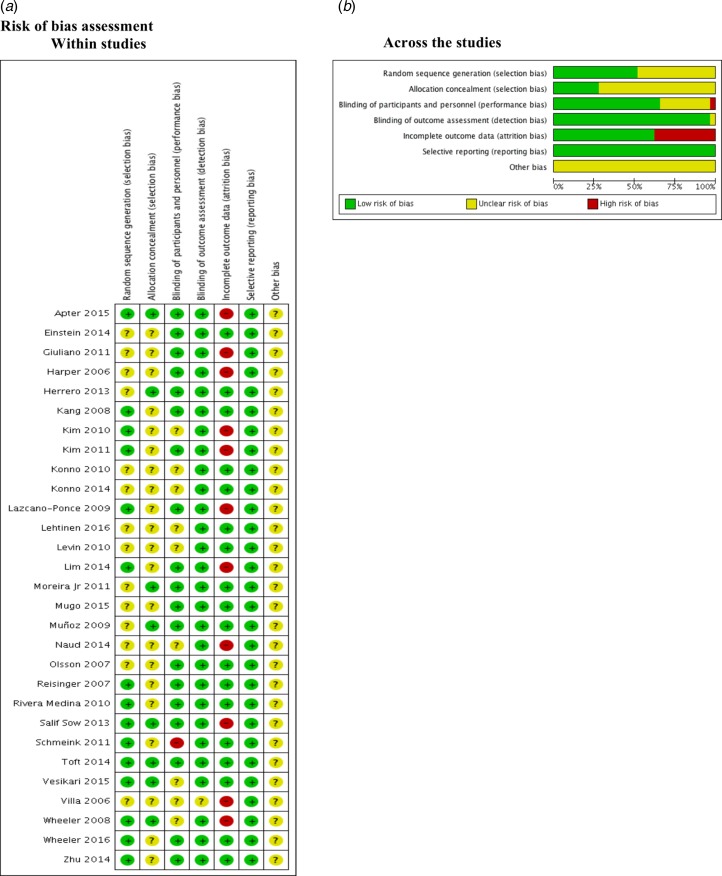
Risk of bias assessment.

No selective reporting was identified during grading in these studies. Nonetheless, all studies were industry-funded, and this leads to unclear classification (Fig. 2).

### Description of effect for comparisons and outcomes

#### Bivalent *vs.* other vaccine/placebo: total

We found significant effect regarding the HPV16, HPV18 and HPV16/18 genotypes. Five studies (Konno, 2014; Zhu, 2014; Harper, 2006; Herrero 2013; Apter, 2015) searched for HPV16 as the outcome, finding a RD −0.05, 95% CI −0.098 to −0.0032 (*I*^2^ = 99%). We found statistical differences and high heterogeneity.

Regarding the HPV18 genotype, we found five studies (Konno, 2014; Zhu, 2014; Harper, 2006; Herrero, 2013; Apter, 2015) which showed statistical differences (RD −0.03, 95% CI −0.062 to −0.0004) (*I*^2^ = 98%). Additionally, regarding HPV16/18 genotype, we included eight studies (Konno 2014; Zhu 2014; Wheeler 2016; Harper 2006; Konno 2010; Naud 2014; Herrero 2013; Apter 2015) showing a RD of −0.1, 95% CI −0.16 to −0.04 (*I*^2^ = 99%).

Other results for any genotype, little adverse effects and autoimmune disease are shown in Table 2.

**Table 2.** Estimated effect of comparisons and outcomes

Outcome	Number of studies	Effect (RD)	95% CI	*I*^2^ (%)
Bivalent *vs.* other vaccine/placebo: total				
Any genotype	3	−0.02	(−0.0617 to 0.0091)	71.20
HPV16	5	−0.05	(−0.0982 to −0.0032)	99.00
HPV18	5	−0.03	(−0.0620 to −0.0004)	98.70
HPV16/18	8	−0.1	(−0.1607 to −0.0399)	99.00
SAE	11	0.0001	(−0.0075 to 0.0077)	19.00
Short-term AE	3	0.0044	(−0.0341 to 0.0428)	0.10
Autoimmune disease	4	0.0006	(−0.0026 to 0.0038)	0.00
Bivalent *vs.* other vaccine/placebo: 1 year				
HPV16/18	2	−0.03	(−0.0404 to −0.0290)	9.70
SAE	5	−0.003	(−0.0122 to 0.0043)	3.90
Short-term AE	2	−0.001	(−0.0410 to 0.0389)	0.00
Autoimmune disease	2	−0.002	(−0.0107 to 0.0059)	0.00
Bivalent *vs.* other vaccine/placebo: 4 years				
HPV16	4	−0.0377	(−0.0640 to −0.0113)	94.00
HPV18	4	−0.0214	(−0.0377 to −0.0051)	91.60
HPV16/18	4	−0.07	(−0.1622 to 0.0149)	99.00
SAE	4	−0.0002	(−0.0104 to 0.0101)	44.00
Tetravalent *vs.* other vaccine/placebo: total				
HPV6	2	−0.05	(−0.0963 to −0.0230)	66.80
HPV11	2	−0.01	(−0.0310 to −0.0085)	0.00
HPV16	2	−0.02	(−0.0630 to 0.0155)	86.80
HPV18	2	−0.02	(−0.0408 to −0.0123)	34.00
HPV16/18	2	−0.09	(−0.3289 to 0.1344)	98.70
HPV6/11/16/18	3	−0.08	(−0.1769 to 0.0123)	97.60
SAE	7	−0.001	(−0.0046 to 0.0024)	11.00
Short-term AE	7	0.02	(−0.0310 to 0.0796)	91.00
Tetravalent *vs.* other vaccine/placebo: 4 years				
HPV6	2	−0.04	(−0.0650 to −0.0281)	0.00
HPV11	2	−0.01	(−0.0310 to −0.0085)	0.00
HPV16	2	−0.06	(−0.1126 to −0.0091)	76.00
HPV18	2	−0.02	(−0.0397 to −0.0114)	0.00
HPV6/11/16/18	2	−0.11	(−0.2035 to −0.0220)	90.00
SAE	2	−0.001	(−0.0066 to 0.0042)	0.00
Short-term AE	2	−0.02	(−0.0766 to 0.0279)	84.00

HPV, human papilloma virus; SAE, serious adverse effects; AE, adverse effects.

#### Bivalent *vs.* other vaccine/placebo: 1 year

Regarding the results at 1 year, we found differences in HPV16/18 genotypes. We included two studies (Herrero, 2013; Apter, 2015) with an RD of −0.0347, 95% CI −0.0404 to −0.0290 (*I*^2^ = 9.7%).

Other results for little adverse effects and autoimmune disease are shown in Table 2.

#### Bivalent *vs.* other vaccine/placebo: 4 year

Regarding the results at four years, we found significant effect regarding the HPV16 and HPV18 and HPV16/18 genotypes. We found four studies (Konno, 2014; Zhu, 2014; Harper, 2006; Herrero, 2013), showing statistical differences with a RD −0.0377, 95% CI −0.0640 to −0.0113 (*I*^2^ = 94.0%) for HPV16 genotype.

Additionally, four studies (Konno, 2014; Zhu, 2014; Harper, 2006; Herrero, 2013) were evaluated for HPV18 genotype. We found a RD −0.0214 (95% CI −0.0377 to −0.0051) (*I*^2^ = 19.6%), showing statistical differences.

Other results for HPV16/18 and small adverse effects are shown in Table 2.

#### Tetravalent *vs.* other vaccine/placebo: total

Regarding this comparison, we found a significant effect in HPV11, HPV6 and HPV18 genotypes. Two studies were compared for HPV6 (Giuliano, 2011; Villa, 2006) showing an RD of −0.0500, 95% CI −0.0963 to −0.0230 (*I*^2^ = 66.8%). For HPV11, two studies were involved in the analysis with an RD −0.0198, 95% CI −0.0310 to −0.0085 (*I*^2^ = 0.00%) (Giuliano, 2011; Villa, 2006). For HPV18 genotype, Giuliano, 2011 and Villa, 2006 were included in the analysis, showing an RD of −0.0200, 95% CI −0.0408 to −0.0123 (*I*^2^ = 34%).

Other results regarding HPV6, HPV16, HPV16/18, HPV6/11/16/18 and small adverse effects are shown in Table 2.

#### Tetravalent *vs.* other vaccine/placebo: 4 years

Regarding the results at four years, we found positive effects in HPV6, HPV11, HPV16, HPV18 and HPV6/11/ 16/ 18 genotypes. For HPV6 two studies were included (Giuliano, 2011; Villa, 2006) with a RD of −0.0465, 95% CI −0.0650 to −0.0281 (*I*^2^ = 0.0%). Two studies (Giuliano, 2011; Villa, 2006) analysed the HPV11 genotype with a RD of −0.0198, 95% CI −0.0310 to −0.0085 (*I*^2^ = 0.0%). For HPV16 and HPV18 genotypes, two studies were included (Giuliano, 2011; Villa, 2006). Regarding the HPV16 genotype, we found a RD of −0.0608, 95% CI −0.1126 to −0.0091 (*I*^2^ = 76.4%) and regarding the HPV18 genotype, the RD was −0.0256, 95% CI −0.0397 to −0.0114 (*I*^2^ = 0.0%). The same studies were included for HPV6/11/16/18 genotype analysis, showing an RD −0.1127, 95% CI −0.2035 to −0.0220.

Other results regarding short-term adverse effects and serious adverse effects are shown in Table 2.

#### Nonavalent *vs.* tetravalent: total

Only one study (Vesikari 2015) compared the 9vHPV against the tetravalent vaccine. The study was conducted in different countries and included people from 9 to 15 years. The outcome was evaluated at 7 months and the results were: anti-HPV16 and anti-HPV18, geometric mean titer (GMT) were similar between vaccines (anti-HPV16 GMTs: 6739.5 *vs.* 6887.4 mMU/ml for nonavalent and tetravalent vaccines and anti-HPV18 GMTs: 1956.6 *vs.* 1795.6 mMU/ml for nonavalent and tetravalent vaccines). For anti-HPV6 and anti-HPV11, GMTs were numerically similar between vaccines (anti-HPV6: 1679.4 *vs.* 1565.9 mMU/ml for nonavalent and tetravalent vaccines and anti-HPV11: 1315.6 *vs.* 1417.3 mMU/ml for nonavalent and tetravalent vaccines).

## Discussion

### Summary of the main results

In summary, evidence suggested that the bivalent HPV vaccine offers protection against HPV16, HPV18 and HPV16/18 genotypes without increasing adverse effects. It is consistent with 4 years for HPV16 and HPV18 genotypes. On the other side, tetravalent HPV vaccine, offered protection against HPV6, HPV11, HPV16 and HPV18 without increasing adverse effects and this was consistent at 4 years.

### Comparison with other reviews or other studies

Detecting a specific capsid antibody in serum suggests an HPV infection. Although more than 50% of infected subjects will have a serological conversion, and thus detection of HPV antibodies. This has been used to analyse the natural history and the cumulative infection in different groups [55].

Assays measuring functional neutralising ability might reflect protective immunity, based on animal experiments. Nonetheless, epitopes for HPV are not completely characterised in human responses therefore, they will be heterogeneous [55].

Neutralisation assays are independent of vaccine material, therefore they provide an unbiased measure of HPV serological response, induced by the vaccine. The direct ELISA, assesses the immunoglobulin G (IgG) response (both antibodies), that might be used as a technique to manage the large volume of samples [56].

On the other hand, the immune response to HPV infection (mainly IgG) is generally weak and heterogeneous among women. Around 50% of the individuals serologically convert to the L1 protein of HPV6, 16 and 18, within 18 months [57]. Conversely, it signifies that more than 40% of women do not seroconvert over time, therefore, the HPV L1 capsid-specific antibody is not an important diagnostic test for this infection. Other HPV antigens (E1, E2, E6 and L2) do not resemble any responses in patients with HPV infection [58]. But, HPV vaccines are based on recombinant virus-like particles (VLPs). These vaccines are highly immunogenic; induce very high titres of neutralising antibodies and represent durable responses. This might be due to VLPs since its ordered structure which permits the presentation of epitopes to B-cells for potent activation [59].

The challenge would be demonstrating that L1 gene expressed via a recombinant virus and the L1 protein produced largely and self-assembled into the VLP or the empty capsid almost identical to the native virion. These VLPs generate neutralising antibodies in animal models and these were protected against a virus challenge [60].

### Strengths and limitations of the review

The advantages of this systematic review include: following standardised methods, according to Cochrane collaboration and PRISMA guidelines and the wide-ranging search to identify data about both clinical and immunological outcomes.

Although we had a lot of studies, most of our limitations were regarding available data for the vaccines across the studies. Additionally, we found differences in study design and methods to assess specific efficacy endpoints and immune responses [61].

On the other hand, we found high statistical heterogeneity and different issues regarding the risk of bias, mainly for not having enough information to evaluate the effect. However, some studies had a high risk of bias regarding blinding and attrition bias. All studies were industry-funded; therefore, this might lead to an unclear risk of bias.

### Implications for policy and research

Hence, this is the first step to evaluate the efficacy of the vaccines against HPV. According to these findings, the vaccines reduce the risk of having the genotype that they were designed for. However, we need to go further, assessing the efficacy to reduce the risk of cancer. Although they were statistically valid, the risk reduction was not high (1–10%) compared with the general chance of not receiving intervention. Therefore, it is essential to consider this when decision-making in public health.

Another interesting data for public health is that there were similar adverse effects in both groups (vaccine *vs.* no intervention), therefore, even if risk reduction was not high, there were no increase of adverse effects in vaccinated people.

## Conclusions

There was a reduction in the prevalence of HPV16, HPV18 and HPV16/18 genotypes when applying the bivalent vaccine, with no increase in adverse effects. Regarding the tetravalent vaccine, we found a reduction in the prevalence of HPV6, HPV11, HPV16 and HPV18 genotypes against placebo/no intervention, with no increase in adverse effects.

We suggest describing better and establishing measures to prevent attrition bias in RCTs regarding this critical issue.

## Supplementary material

For supplementary material accompanying this paper visit https://doi.org/10.1017/S0950268818003679. click here to view supplementary material
