# Supplementary material for: The effectiveness of vaccination to prevent the papillomavirus infection: a systematic review and meta-analysis
Source: Epidemiol Infect. 2019 Mar 20;147:e156. doi: 10.1017/S0950268818003679 (PMC6518793; doi:10.1017/S0950268818003679)
Supplement: Supplementary file 1 [file S0950268818003679sup001.docx]

**Search Strategy**

**Medline (Ovid):**

exp Papillomavirus Infections/

exp Papillomaviridae/

Papillomavirid*.mp/

HPV*.mp.

human papillomavirus*.mp.

human papilloma virus*.mp.

or/

exp Papillomavirus Vaccines/

(Papillomavirus adj2 Vaccines).mp

gardasil.mp.

cervarix.mp.

vaccin*.mp.

immuni*.mp.

Or/

randomized controlled trial.pt

controlled clinical trial.pt

randomized.ab

placebo.ab

randomly.ab

trial.ab

(clinical adj2 trial).mp.

(randomi*ed adj2 controlled adj2 trial).mp.

exp double-blind method

or/

**Embase:**

'papillomavirus infection'/exp

(papillomavirus next/2 infection):ti,ab

'papillomaviridae'/exp

papillomavirid*:ti,ab

HPV*:ti,ab

human papillomavirus*:ti,ab

(human next/2 papillomavirus*):ti,ab

human papilloma virus*:ti,ab

or/

'wart virus vaccine'/exp

(Papillomavirus next/2 Vaccine*):ti,ab

gardasil:ti,ab

cervarix:ti,ab

vaccin*:ti,ab

immuni*:ti,ab

Or/

'randomized controlled trial'/exp

(randomi*ed NEXT/2 controlled NEXT/2 trial):ti,ab

'clinical trial'/exp

(clinical NEXT/2 trial):ti,ab

'double blind procedure'/exp

or/

**Central (Ovid)**

exp Papillomavirus Infections/

exp Papillomaviridae/

Papillomavirid*.mp/

HPV*.mp.

human papillomavirus*.mp.

human papilloma virus*.mp.

or/

exp Papillomavirus Vaccines/

(Papillomavirus adj2 Vaccines).mp

gardasil.mp.

cervarix.mp.

vaccin*.mp.

immuni*.mp.

Or/

**HTA; DARE; Cochrane DB SR; ACP journal club; Cochrane methodology register; NHS Economic evaluation database.**

exp Papillomavirus Infections/

exp Papillomaviridae/

Papillomavirid*.mp/

HPV*.mp.

human papillomavirus*.mp.

human papilloma virus*.mp.

or/

exp Papillomavirus Vaccines/

(Papillomavirus adj2 Vaccines).mp

gardasil.mp.

cervarix.mp.

vaccin*.mp.

immuni*.mp.

Or/

randomized controlled trial.pt

controlled clinical trial.pt

randomized.ab

placebo.ab

randomly.ab

trial.ab

(clinical adj2 trial).mp.

(randomi*ed adj2 controlled adj2 trial).mp.

exp double-blind method

or/
